# Dual PTP1B/TC-PTP Inhibitors: Biological Evaluation of 3-(Hydroxymethyl)cinnoline-4(*1H*)-Ones

**DOI:** 10.3390/ijms24054498

**Published:** 2023-02-24

**Authors:** Kira V. Derkach, Maxim A. Gureev, Anastasia A. Babushkina, Vladimir N. Mikhaylov, Irina O. Zakharova, Andrey A. Bakhtyukov, Viktor N. Sorokoumov, Alexander S. Novikov, Mikhail Krasavin, Alexander O. Shpakov, Irina A. Balova

**Affiliations:** 1 Sechenov Institute of Evolutionary Physiology and Biochemistry, Russian Academy of Sciences, Thorez av. 44, 194223 St. Petersburg, Russia; 2 Center of Bio- and Chemoinformatics, I.M. Sechenov First Moscow State Medical University, 119991 Moscow, Russia; 3 Institute of Chemistry, Saint Petersburg State University, Universitetskaya nab. 7/9, 199034 St. Petersburg, Russia; 4 Institute for Medicine and Life Sciences, Immanuel Kant Baltic Federal University, 236041 Kaliningrad, Russia

**Keywords:** cinnolines, phosphatase inhibitors, obesity, T-cell tyrosine phosphatase, tyrosine phosphatase 1B, insulin, leptin

## Abstract

Dual inhibitors of protein phosphotyrosine phosphatase 1B (PTP1B)/T-cell protein phosphotyrosine phosphatase (TC-PTP) based on the 3-(hydroxymethyl)-4-oxo-1,4-dihydrocinnoline scaffold have been identified. Their dual affinity to both enzymes has been thoroughly corroborated by in silico modeling experiments. The compounds have been profiled in vivo for their effects on body weight and food intake in obese rats. Likewise, the effects of the compounds on glucose tolerance, insulin resistance, as well as insulin and leptin levels, have been evaluated. In addition, the effects on PTP1B, TC-PTP, and Src homology region 2 domain-containing phosphatase-1 (SHP1), as well as the insulin and leptin receptors gene expressions, have been assessed. In obese male Wistar rats, a five-day administration of all studied compounds led to a decrease in body weight and food intake, improved glucose tolerance, attenuated hyperinsulinemia, hyperleptinemia and insulin resistance, and also compensatory increased expression of the PTP1B and TC-PTP genes in the liver. The highest activity was demonstrated by 6-Chloro-3-(hydroxymethyl)cinnolin-4(*1H*)-one (compound **3**) and 6-Bromo-3-(hydroxymethyl)cinnolin-4(*1H*)-one (compound **4**) with mixed PTP1B/TC-PTP inhibitory activity. Taken together, these data shed light on the pharmacological implications of PTP1B/TC-PTP dual inhibition, and on the promise of using mixed PTP1B/TC-PTP inhibitors to correct metabolic disorders.

## 1. Introduction

Protein phosphotyrosine phosphatase 1B (PTP1B) is a negative regulator of metabolic pathways activated by insulin (which is produced by pancreatic beta cells) and by adipokine leptin (which is produced by adipose tissue) [1,2]. In response to insulin stimulation, PTP1B dephosphorylates the active phosphorylated forms of the insulin receptor and insulin receptor substrate proteins-1 and -2 (IRS-1, IRS-2) [3,4]. Under leptin stimulation, the enzyme dephosphorylates the leptin-activated non-receptor Janus kinase 2 (JAK2) and IRS1/2-proteins, the key components of leptin signaling [2,5]. Suppression of PTP1B phosphatase activity abolishes its inhibitory effect on insulin and leptin signaling. In obesity, type 2 diabetes mellitus (T2DM), and metabolic syndrome, the insulin and leptin signaling pathways are attenuated as a result of long-term exposure to hyperinsulinemia and hyperleptinemia; thus, the use of PTP1B inhibitors may become one of the approaches to restore them and increase tissue sensitivity to insulin and leptin [1,6]. PTP1B can be inhibited by small molecules targeting either the catalytic or the allosteric site of the enzyme. However, inhibitors aimed at the allosteric site are expected to exert more specific inhibition because the allosteric site of PTP1B, unlike its catalytic site, is more distinct from the allosteric sites of other PTP1B-related phosphatases, such as the T-cell protein phosphotyrosine phosphatase (TC-PTP) and endothelial Src homology region 2 domain-containing phosphatase-1 (SHP1) [1,6,7]. TC-PTP is also capable of attenuating the insulin and leptin signaling pathways by dephosphorylating their receptor and post-receptor components [8,9]. TC-PTP inhibitors, similarly to PTP1B inhibitors, can also improve glucose homeostasis and prevent obesity in metabolic syndrome and T2DM [10].

Previously, selective PTP1B phosphatase inhibitors have been developed [11,12,13,14], which had little effect on TC-PTP activity. The therapeutic potential of such inhibitors may not be fully justified. Inhibition of PTP1B alone can lead to undesirable toxic effects, which have been observed in previous in vivo experiments [15] and, similarly, can cause a compensatory increase in the activity of other phosphatases, primarily TC-PTP, which can functionally replace PTP1B [16,17,18,19]. Consequently, currently, there are no approved PTP1B inhibitors [20], despite the high demand for the effective treatment of the diseases associated with insulin and leptin resistance (T2DM, metabolic syndrome, and obesity). Accordingly, a paradigm shift might be needed to create such inhibitors, which would act on both PTP1B and TC-PTP, though not necessarily with high potency.

The aim of the present work is to identify small molecule inhibitors that would potentially be able to suppress the functional activity of both tyrosine phosphatases, PTP1B and TC-PTP, and thereby affect the metabolic and hormonal parameters in rats with diet-induced obesity.

**Figure 1:**
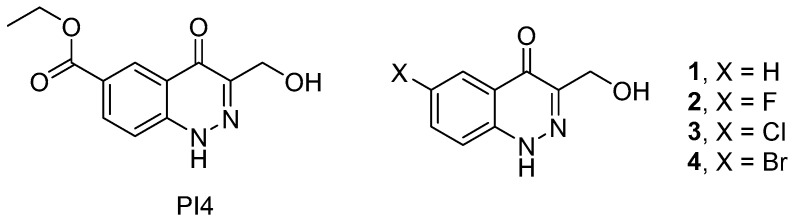
3-(Hydroxymethyl)-4-oxo-1,4-dihydrocinnoline derivatives investigated earlier (PI4) and reported in this work (**1**–**4**).

As a lead structure in our quest for dual PTP1B/TC-PTP inhibitors, we relied on the earlier reported compound PI4 (ethyl 3-(hydroxymethyl)-4-oxo-1,4-dihydrocinnoline-6-carboxylate), a 4-oxo-1,4-dihydrocinnoline derivative, that demonstrated an inhibitory activity towards PTP1B, and exerted a stimulating effect on components of the insulin and leptin signaling pathways in rat hypothalamic neurons, including the serine/threonine protein kinase Akt and transcription factor STAT3 (signal transducer and activator of transcription 3) [21]. In addition, PI4 reduced the body and fat weights in diet-induced obese rats, suppressed their food intake, improved metabolic parameters, and increased their sensitivity to insulin and leptin [22]. In this study, we hypothesized that by exploring an expanded set of 3-(hydroxymethyl)-4-oxo-1,4-dihydrocinnoline analogs **1**–**4** (Figure 1), we might not only obtain yet another set of PTP1B inhibitors but could potentially identify dual inhibitors of PTP1B/TC-PTP, which are sought after in the context of metabolic disease treatments (vide supra).

## 2. Results

### 2.1. Compounds ***1***–***4***

Compounds **1**–**4** were obtained, as described previously [23].

### 2.2. Inhibitory Effects of Compounds ***1***–***4*** towards PTP1B and TC-PTP

**Figure 2:**
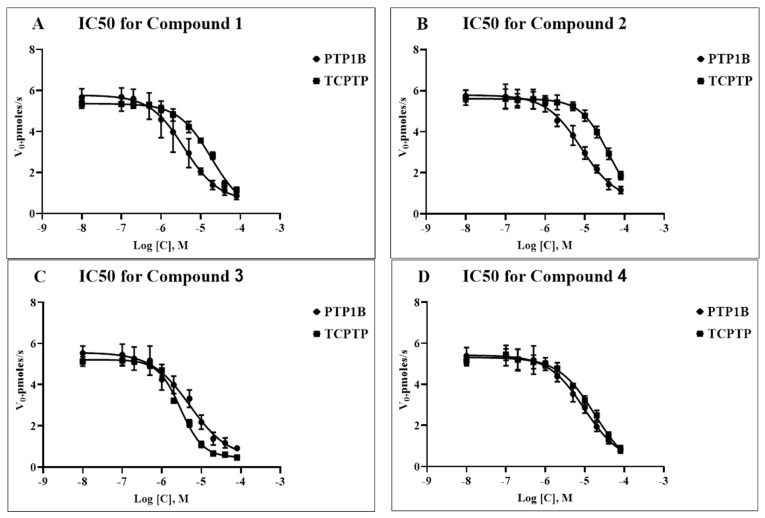
Dose-response curves *^a^* of PTP1B and TC-PTP inhibition by compounds **1** (**A**), **2** (**B**), **3** (**C**), and **4** (**D**). The *X*-axis represents the logarithm of the concentration of the tested compound (in moles/L), while the *Y*-axis represents the initial velocities of the enzymatic reactions (in pmoles/s), respectively, for PTP1B (black circles) and TC-PTP (black squares).

Compounds **1**–**4** displayed dose-dependent inhibition of both phosphatases, PTP1B and TC-PTP, as expressed in the IC_50_ values (Table 1) calculated from the respective dose-response curves (Figure 2). In each case, the range from 0.1 to 80 μM of the tested compound was studied. As can be seen from Table 1, compounds **1** and **2** showed a ~5–7-fold higher selectivity for PTP1B compared to that for TC-PTP, and in the case of compound **2**, the differences were significant (*p* = 0.001). In the case of compounds **3** and **4**, the IC_50_ values for PTP1B and TC-PTP were comparable, indicating their close selectivity for both enzymes. In the case of compound **3**, the IC_50_ value for TC-PTP was significantly lower than the IC_50_ values for TC-PTP inhibition by compounds **1** and **2** (*p* = 0.01 and *p* < 0.0001, respectively), although they did not differ significantly from that of compound **4** (*p* = 0.12), which indicates a more pronounced TC-PTP inhibitory effect of compound **3** as compared to compounds **1** and **2**. In the case of PTP1B, no significant differences in the IC_50_ values were found between all the studied compounds. The obtained IC_50_ values for compounds **1**–**4** were close to the IC_50_ values for a number of natural tyrosine phosphatase inhibitors and their semisynthetic derivatives [24,25,26,27], which may indicate a similar dose dependence to their inhibitory effect on the activity of PTP1B and TC-PTP. At the same time, compounds **1–4** had higher IC_50_ values in comparison with those for some recently developed synthetic phosphatase inhibitors, yet they were inferior to them in their binding affinity to the allosteric sites of these enzymes [18,19].

### 2.3. The Effects of Compounds ***1***–***4*** on the Body Weight, Food Intake, Glucose Tolerance and Insulin and Leptin Levels in the Blood of Male Rats with HFD-Induced Obesity

Based on the results of the in vitro experiments and our earlier data on the pharmacodynamics of compound PI4 [21,22], we studied the anorexigenic effect of compounds **1**–**4** and their influence on glucose homeostasis, as well as insulin and leptin resistance, in rats with diet-induced obesity.

**Figure 3:**
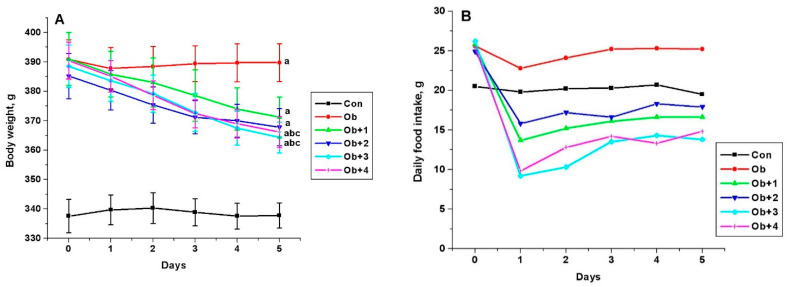
The body weight (**A**) and the consumption of a standard food mixture (**B**) in control and obese male Wistar rats, and the effect on them after treatment with compounds **1**–**4**. Obese rats received phosphatase inhibitors (in DMSO, 300 µL) for 5 days (the compounds **1** and **2** at a daily dose of 7 mg/kg, and the compounds **3** and **4** at the daily doses of 8 and 10 mg/kg, respectively). Control and obese rats received DMSO, solvent for phosphatase inhibitors, instead of the tested compounds. Six groups of animals were studied: control (Con, n = 10), obesity (Ob, n = 10), obesity with five-day treatment with compounds **1** (Ob + 1, n = 5), **2** (Ob + 2, n = 5), **3** (Ob + 3, n = 10), and **4** (Ob + 4, n = 10). The body weight and food intake were assessed before (point 0) and during 5 days of treatment. In Figure 3A: ^a^—the difference to the control animals is significant at *p* < 0.05; ^b^—the difference to the untreated obese rats (Ob) is significant at *p* < 0.05; ^c^—difference between body weight before and after treatment of obese rats with the studied compounds is significant at *p* < 0.05. *M ± SEM*, n = 5 (Ob + 1, Ob + 2) and n = 10 (Con, Ob, Ob + 3, Ob + 4).

The five-day treatment of obese male rats with all the studied compounds (**1** and **2** at a dose of 7 mg/kg/day, and **3** and **4** at doses of 8 and 10 mg/kg/day, respectively) led to a decrease in the body weight and food consumption. This indicates a pronounced anorexigenic effect of compounds **1**–**4**. The highest effect was demonstrated by compounds **3** and **4**, which reduced the body weight in obese rats by 22.2 ± 4.5 and 22.6 ± 4.0 g, respectively, (*p* < 0.05 as compared to untreated obese animals) and reduced the consumption of dry standard food by 46% and 39%, respectively (Figure 3).

**Figure 4:**
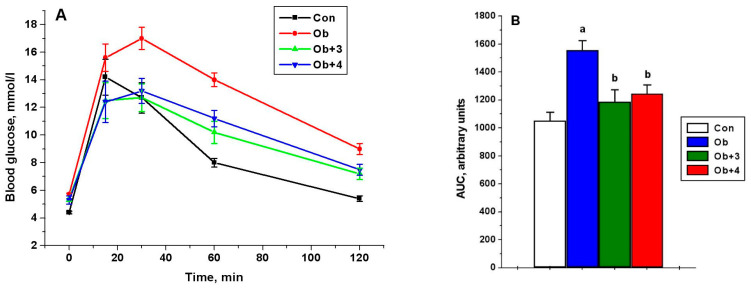
The concentration of glucose in the blood of control and obese rats during the test with glucose load and the effect of treatment with compounds **3** and **4** (**A**), as well as the AUC_0-120_ values for glucose concentration curves (**B**). Obese rats received phosphatase inhibitors (in DMSO, 300 µL) for 5 days, such as compound **3** at a daily dose of 8 mg/kg and compound **4** at a daily dose of 10 mg/kg. Control and obese rats received DMSO, instead of the tested compounds. Four groups of animals were studied: control (Con), obesity (Ob), obesity with the treatment with compounds **3** (Ob + 3) and **4** (Ob + 4). For the glucose tolerance test, five animals were taken from each group, and the glucose levels were assessed before and after 15, 30, 60 and 120 min after the glucose load. For glucose concentration curves, the AUC_0-120_ values were calculated as an integrative indicator of blood glucose levels within 120 min after glucose administration. The data are presented as *M ± SEM*, n = 5. In Figure 4B: ^a^—the difference to the control rats is significant at *p* < 0.05; ^b^—the difference to the untreated obese rats (Ob) is significant at *p* < 0.05.

Compounds **1**–**4** had no significant effect on glucose levels, although, of the most active compounds (**3** and **4**), compound **4** improved the glucose sensitivity, previously reduced in obesity, as shown by the glucose tolerance test. This was indicated by the values of the glucose levels 120 min after glucose load, which were reduced by 20% and 17% in the groups treated with compounds **3** and **4**, respectively, in comparison with the untreated obese animals, as well as the values of AUC_0-120_ for the curves “glucose concentration (mM)”—time (minutes)”. The AUC_0-120_ values in the groups treated with compounds **3** and **4** were reduced by 24% and 20%, respectively, as compared with the untreated animals (*p* < 0.05 as compared to the Ob group) (Figure 4).

The levels of Insulin and leptin were elevated in obese rats, indicating the development of insulin and leptin resistance. Measurement of the levels of insulin and leptin in the blood of animals, before and after the treatments with compounds **1**–**4**, showed that the compounds caused a decrease in the levels of these hormones (except for the insulin level in the Ob + **2** group), which indicates an increase in the sensitivity to insulin and leptin. The inhibiting effect on insulin and leptin levels exerted by compounds **3** and **4** was more pronounced compared to compounds **1** and **2**. For the purpose of normalization, insulin sensitivity was indicated by the index of insulin resistance (IR), calculated as the product of the blood concentrations of glucose and insulin. In the group treated with compound **3**, the insulin resistance index was significantly reduced compared to the Ob group, yet did not differ from that in the control animals (Table 2).

### 2.4. The Effects of Compounds ***1***–***4*** on Gene Expression in the Livers of Rats with HFD-Induced Obesity

Based on the in vitro IC_50_ values for PTP1B and TC-PTP inhibition by compounds **1**–**4**, and on their ability to increase insulin sensitivity and reduce hyperleptinemia, established in the in vivo experiments, we studied the effect of these compounds on the expression of the genes encoding PTP1B and TC-PTP, as well as for insulin and leptin receptors, in the livers of obese rats. Along with this, we studied the expression of cytoplasmic tyrosine phosphatase SHP1, the catalytic site of which differs from those of PTP1B and TC-PTP [28].

The treatments with all the studied compounds led to an overall increase in the expression of the *PTP1B* and *TCPTP* genes in the liver. However, a significant difference to the Ob group was shown only for the groups treated with compounds **3** and **4**, and, in the case of the *TCPTP* gene, for the group treated with compound **1**. Compounds **3** and **4** did not affect the expression of the *Shp1* gene, which encodes the protein tyrosine phosphatase SHP1, while compounds **1** and **2** increased the expression of the *Shp1* gene, and the differences from the control were significant. These data suggest that the unsubstituted and fluoro-substituted analogs, **1** and **2**, are likely to also inhibit phosphatase SHP1.

**Figure 5:**
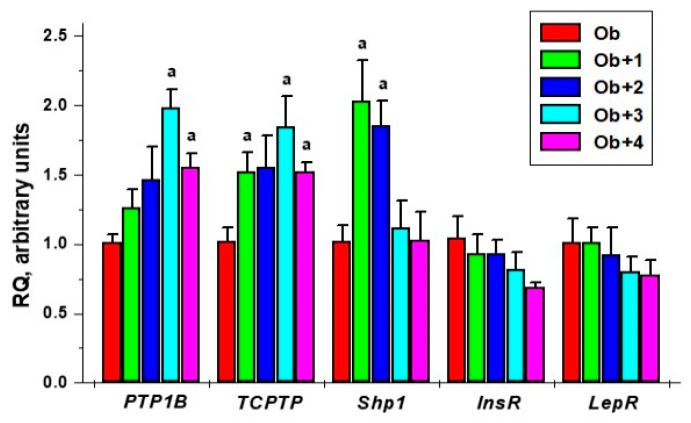
The effect of the five-day treatment of obese rats with compounds **1**–**4** on the gene expression of the tyrosine phosphatases, such as PTP1B, TC-PTP, and SHP1, and the insulin and leptin receptors in the liver. ^a^—the difference with untreated obese rats is significant at *p* < 0.05. *M* ± *SEM*, n = 5.

Compounds **3** and **4** caused a slight decrease in the expression of the genes encoding the insulin and leptin receptors, but the difference with the Ob group was not significant. This tendency, albeit rather weak, could be due to an increase in the sensitivity of hepatocytes to insulin and leptin (Figure 5).

### 2.5. Docking Simulation of Compounds ***1***–***4*** as PTP1B/TC-PTP Inhibitors

The evaluation of the specific biological activity of compounds **1**–**4** showed their capability to interact with PTP1B and TC-PTP. Based on the gene expression data of phosphatases in the liver, there are reasons to believe that compounds **1** and **2** are also potentially able to interact with the SHP1 phosphatase, which has a catalytic site different from that of other closely related phosphatases, PTP1B and TC-PTP. It is necessary to establish the causes of this phenomenon to reduce the spectrum of side interactions.

**Figure 6:**
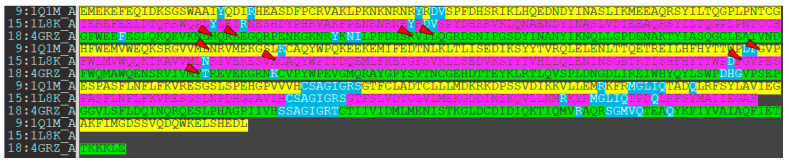
The amino acid residues of phosphatases participating in their interaction with low-molecular ligands (highlighted by blue color). Green: PTP1B (1Q1M), magenta: TC-PTP (1L8K), and yellow: SHP-1 (4GRZ). Red arrows show amino acid residues, excluded from enzyme–ligand interactions due to protein model structural changes.

As a first step, we studied the active sites of the phosphatases PTP1B, TC-PTP, and SHP1. The amino acids that form the catalytic sites of the enzymes are shown in Figure 6 and are highlighted in blue.

As can be seen from Figure 6, some identical amino acid residues are not included in the catalytic site formation.

**Figure 7:**
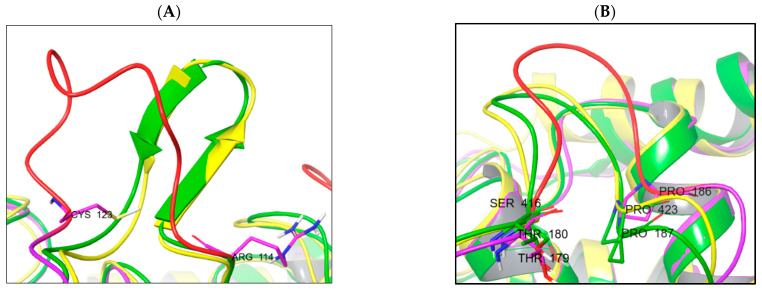
Structural differences in enzyme structure between PTP1B (yellow), TC-PTP (magenta/red), and SHP-1 (green). (**A**) Comparison of Arg114–Cys123 domain spatial differences and secondary structures; (**B**) domain Thr179–Pro186 spatial orientation.

The main reason is the difference in the protein loop conformations and the presence of secondary structure elements. The TC-PTP enzyme is less structured in the domain between Arg114 and Cys123 (unstructured loop). This domain in PTP1B (Arg114–Cys123) and SHP-1 (Arg352–Cys361) is more rigid due to the β-sheet secondary structure *element presence* (Figure 7A). An unstructured loop in the case of TC-PTP can be useful for studies into ligand-induced conformational changes. The second domain, very different in the studied phosphatases, is located between the following residues: Thr180–Pro187 (PTP1b), Thr179–Pro186 (TC-PTP), and Ser416–Pro423 (SHP-1) (Figure 7B).

Primarily, in the PTP1b and TC-PTP structures, this domain is identical by sequence (TWPDFGVP), but in the SHP-1 phosphatase, it is different (SWPDHGVP). The main ligand-interacting sequences «WPD» and «GVP» are conserved, although the phenylalanine residue is changed to histidine and the threonine residue is replaced by serine. The substitution of phenylalanine for histidine provides a decrease in the efficiency of the hydrophobic interactions and an increase in the efficiency of polar interactions (also pH-dependent), in this part of the active site of the enzymes.

**Figure 8:**
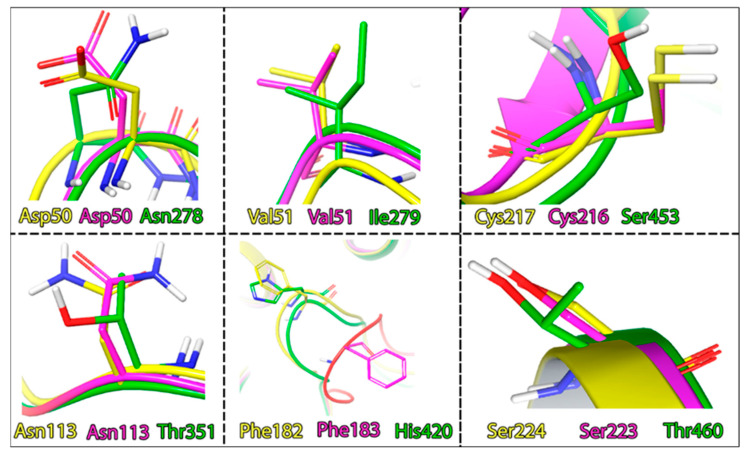
Differences in the amino acid composition of the active sites of the phosphatases, coloring indicates, PTP1B: yellow; TC-PTP: magenta; SHP1: green.

If we compare the geometry of the TC-PTP and PTP1B loops, we will identify significant differences in their positions. However, their sequences remain the same. The key differences, here, are hidden in the different rotamer states of the Phe–Asp pair. Another difference lies in the amino acid properties forming the active site cavity. Here, we can observe the difference in the interacting amino acids profile: SHP1 is less hydrophobic and more polar with the addition of a histidine residue. The homology degree in these segments of PTP1B and TC-PTP is also much higher (Figure 8).

**Figure 9:**
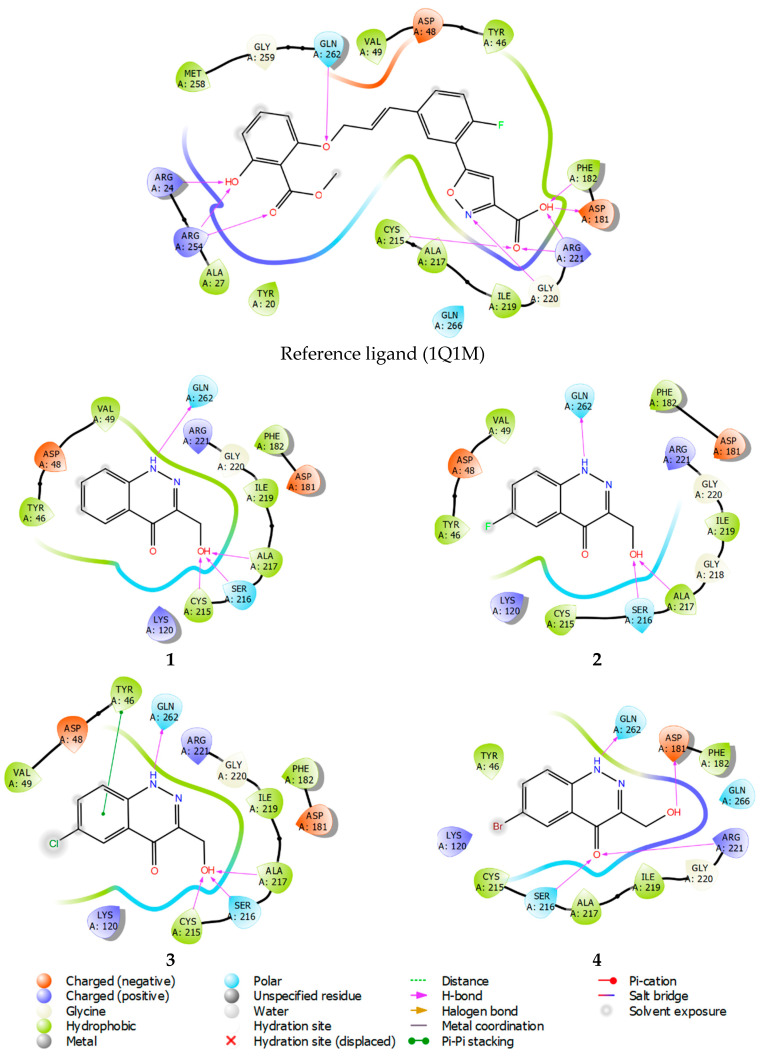
Ligand interaction diagrams of observed ligands with PTP1B.

Molecular docking results and the binding free energies of compounds **1**–**4** are shown in Table 3. Studied compounds bind stably within the active site of all the observed phosphatases. With regard to PTP1B, compounds **1**–**4** can be considered site-specific ligands, because the ligand efficiency (LE) value (Table 3) is superior to that of the structure of the reference compound. In contrast to the used reference compound, the active inhibitor co-crystallized with PTP1B (pdb model 1Q1M, ligand structure, Figure 9). At the same time, a binding mode for the studied structures **1**–**4** also reproduces interactions specific to the reference compound (see Figure 9, PTP1B inhibitor showed in PDB model 1Q1M).

When switching to the alternative targets of TC-PTP and SHP1, we observe a decrease in the active site specificity. At the same time, in the case of SHP1, the compounds also change the binding area (which remains within the active site). Compounds **1** and **2** retain high levels of site-specificity towards SHP1 (Table 3—highlighted by orange). Conversely, compounds **3** and **4** show a potential capability to selectively interact with PTP1B and TC-PTP (Table 3—highlighted by green). More clearly, the specificity of the observed targets is corroborated by the free energy value (ΔG). Such significant differences in comparison to the docking results, in theory, may indicate a significant solvent role in the binding process (the MM-GBSA method considers this, implicitly). The binding selectivity to PTP1B and TC-PTP for compounds **3** and **4** agrees with our experimental data.

Ligand interaction diagrams analysis, presented in Figure 9, shows that compounds **1**–**4** interact mainly within the amino acids: Asp181, Arg221, Phe182, Cys215, and Ala217, in the same manner as the reference compound. Thus, compounds **1–4** mimic the key lipophilic and polar contacts, from which PTP1B site-specificity is achieved.

The diagrams of the ligand–protein (enzyme) interactions with TC-PTP were similarly analyzed.

**Figure 10:**
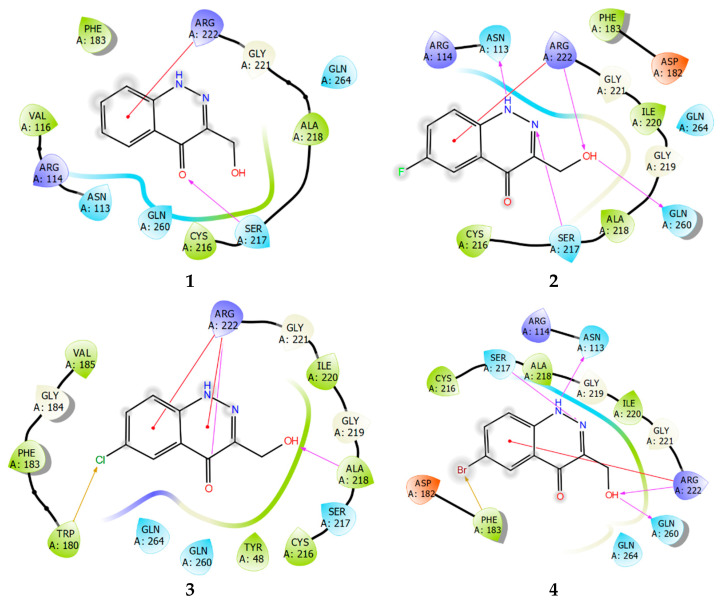
Ligand interaction diagrams of observed ligands **1**–**4** with TC-PTP.

Studying the protein–ligand interactions of compounds **1**–**4** with TC-PTP showed that the interaction profile for these compounds is almost identical. A distinctive feature is the weakening of the network of lipophilic ligand–enzyme contacts in the cavity of the active site. Moreover, unlike PTP1B, a pi–cation interaction with Arg222 is realized (Figure 10). In the TC-PTP structure, it is more accessible for any subsequent interaction.

**Figure 11:**
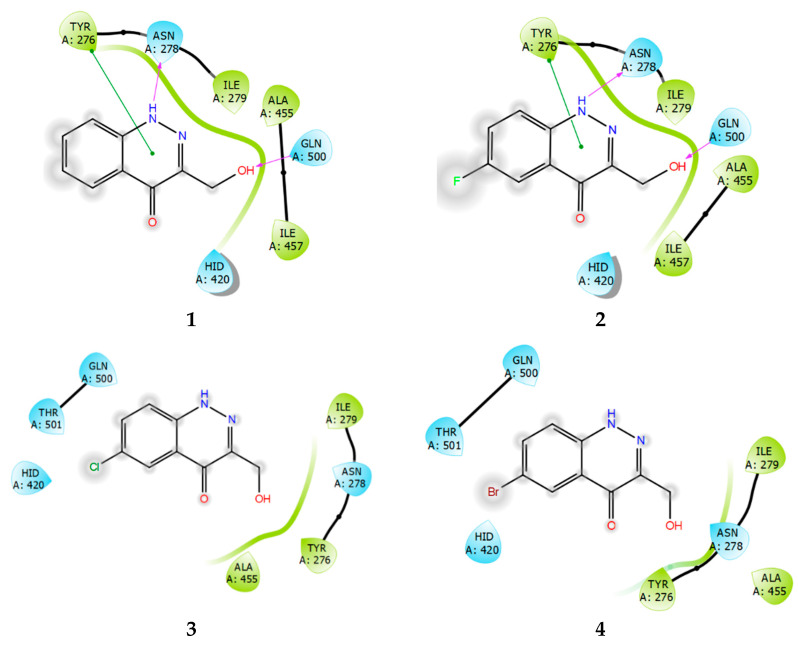
Ligand interaction diagrams of observed ligands **1**–**4** with SHP1.

Ligand interaction diagrams of compounds **1**–**4** with SHP1 (Figure 11) showed that these compounds are more than capable of interacting with the enzyme, to form a stable protein–ligand complex. However, the binding region is strongly shifted towards Tyr276, relative to the binding site. Both PTP1B and TC-PTP contain the residues Tyr46 and Tyr48, which have a similar arrangement in each phosphatase molecule. In regard to the reference structure of the PTP1B ligand (Figure 9), then, Tyr46 interacts with the lipophilic fluorophenyl part of the molecule, sometimes forming a pi–stacking interaction. Compounds **3** and **4** are distinguished by a lower intensity of lipophilic contacts with SHP1 and by the absence of the pi–stacking interaction with Tyr276 (Figure 11).

## 3. Discussion

Our in vitro data indicate that all 4-oxo-1,4-dihydrocinnoline derivatives **1**–**4**, which are structural analogs of the previously studied compound PI4 [21,22], are inhibitors of the PTP1B and TC-PTP tyrosine phosphatases. At the same time, they differ in selectivity for these tyrosine phosphatases since compounds **1** and **2** were found to be more selective towards PTP1B, while compounds **3** and **4** had no significant differences in selectivity for either phosphatase. In addition, compound **3** was far more effective as an inhibitor of TC-PTP than compounds **1** and **2** (Table 1). These observations are supported by the molecular docking results.

As noted above, unlike compounds **1** and **2**, compounds **3** and **4**, according to the results of the in vitro experiments, are similar in their ability to inhibit the PTP1B and TC-PTP phosphatases. Notably, the inhibitory effect for PTP1B, as judged by the IC_50_ values, did not fully correlate with the performance of the compounds in the in vivo experiments. Thus, the IC_50_ value for compound **1** was the lowest among all the derivatives studied and was 2.5 times inferior to that for compound **4.** At the same time, the anorexigenic effect of compound **1** was less pronounced compared to compounds **3** and **4**. Importantly, the effects of compounds **3** and **4** on the food intake and metabolic parameters in obese rats are similar, while, according to the in vitro experiments, the effectiveness of compound **3** as an inhibitor of TC-PTP is more pronounced. It can be assumed that the similar affinity of compounds **3** and **4**, with respect to both phosphatases PTP1B and TC-PTP, is important for the metabolic effects of these inhibitors. This distinguishes compounds **3** and **4** from compounds **1** and **2**, which are more selective for PTP1B.

The PTP1B and TC-PTP phosphatases, being negative regulators of insulin and leptin signaling, are involved in the development of insulin and leptin resistance and mediate an increase in appetite, and the accumulation of excess adipose tissue in metabolic disorders [1,6,8,9,10]. At the same time, there are a number of common downstream targets for PTP1B and TCPTP, which makes them at least partly interchangeable. For instance, both phosphatases dephosphorylate the hormone-activated phosphorylated forms of the leptin and insulin receptors, as well as the non-receptor-associated JAK2-tyrosine kinase associated with the leptin receptor [6,10]. However, there are also significant differences in the intracellular targets of the PTP1B and TCPTP phosphatases. The PTP1B dephosphorylates the IRS1/2 proteins that couple the insulin receptor to downstream SH2 domain-containing proteins [3,6], while the TCPTP dephosphorylates the STAT3 transcription factor, which is activated via the leptin receptor and controls the expression of STAT3-dependent genes [10]. The fact that compound **3**, which is the most active with respect to TC-PTP, significantly reduces leptin levels is likely due to the fact that phosphatase TC-PTP is even more involved in the regulation of leptin signaling than in the regulation of insulin signaling [10].

Our results on the high efficiency of the dual inhibitors of the PTP1B and TCPTP phosphatases are supported by numerous studies, whereby a pronounced anorexigenic effect of low-selective inhibitors of PTP1B and TC-PTP was identified, although this phenomenon remains poorly understood. Celastrol, a naturally occurring pentacyclic triterpene, when administered to mice, reduced the activity of both phosphatases in the hypothalamic arcuate nucleus, through an allosteric mechanism, significantly reducing their food intake and body weights. The anorexigenic effects of Celastrol are mainly due to the activation of leptin signaling in hypothalamic neurons [29]. Simultaneous knockout of the PTP1B and TC-PTP genes in the hypothalamus of obese mice, as well as the combined administration of the PTP1B inhibitor and the glucocorticoid hormone antagonist RU486, which attenuates TC-PTP expression, suppressed food intake, normalized body weight and adipose tissue, improved glucose tolerance, alongside insulin and leptin sensitivity [9]. At the same time, inhibiting the two phosphatases separately was significantly less effective. Thus, simultaneous, and similarly effective inhibition of both PTP1B and TC-PTP, which we have shown for 4-oxo-1,4-dihydrocinnolines **1**–**4**, primarily for compounds **3** and **4**, does not allow for the compensatory switching of the mechanisms, from one phosphatase to another, in the inhibition of leptin and insulin signaling. However, one cannot exclude the involvement in these compensatory mechanisms of additional phosphatases, which are less specific in their targeting of the insulin and leptin receptors and their downstream signaling proteins.

Pharmacological or genetic suppression of PTP1B and TC-PTP activity can trigger a number of compensatory mechanisms, which weaken the effects of the inhibitors of these enzymes. Among them, are changes in gene expression of both phosphatases and the components of the insulin and leptin signaling cascade. In the liver, we studied the expression of the genes that encode PTP1B, TC-PTP, and SHP1, as well as genes encoding insulin and leptin receptors. It was shown that in the liver of rats treated with compounds **3** and **4**, the expressions of the PTP1B and TC-PTP genes were significantly increased, while the expression of the *Shp1* gene did not change. In the case of compounds **1** and **2**, which are more specific to PTP1B, there was a trend towards an increase in the PTP1B and TC-PTP gene expressions, yet there was a significant increase in the *Shp1* gene expression. An increase in the expression of the SHP1 phosphatase, in obese rats with impaired glucose tolerance, is consistent with its negative role in the regulation of feeding behaviors and glucose homeostasis [30]. Thus, it was found that in mice with diet-induced obesity, the expression of SHP1 is increased [31], and inhibition or knockout of this enzyme prevents the development of metabolic disorders [30,32]. Thus, there is reason to believe that this phosphatase may be involved in the mechanisms through which insulin and leptin signaling is weakened. Our data on the expression of the SHP1 gene, however, seem somewhat unexpected and require further study. Based on these data, it can be concluded that inhibitors with comparable selectivity towards the PTP1B and TC-PTP phosphatases (compounds **3** and **4**) have little effect on the expression of SHP1, while inhibitors predominantly selective for PTP1B (compounds **1** and **2**), increase it, and this effect does not depend on the IC_50_ values.

## 4. Materials and Methods

### 4.1. Compound Synthesis

Compounds **1–4** were synthesized, as described previously [23].

### 4.2. Biology Studies

#### 4.2.1. The Phosphatases Activity Assay and the Determination of IC_50_ for Tested Compounds

The measurement of the activity of the phosphatases PTP1B and TC-PTP and their inhibition was carried out using 6,8-difluoro-4methylumbelliferyl phosphate (DiFMUP), as previously described [33]. The stock solution of recombinant human PTP1B protein (#ab51277, Abcam, Cambridge, UK), at a concentration of 1 µg/µL, was prepared in 25 mM Tris–HCl (pH 7.5), 20% glycerol, 2 mM β-mercaptoethanol, 1 mm EDTA, and 1 mM DTT, and stored at −20 °C. Active human recombinant TC-PTP protein was purchased from Sigma-Aldrich (#SRP0218, Saint Louis, MO, USA) as the aqueous buffer solution at a concentration of 2.9 mg/mL, and aliquots were stored at −80 °C. The fluorogenic substrate DiFMUP (#D6567, Molecular Probes, Thermo Fisher Scientific, Waltham, MA, USA) was dissolved at a concentration of 10 mM in *N,N*-dimethylformamide and stored in aliquots at −20 °C. The 6,8-difluoro-7-hydroxy-4-methylcoumarin (DiFMU, #D6566, Molecular Probes, Thermo Fisher Scientific, Waltham, MA, USA) was used as a reference fluorescent standard. For the IC_50_ determination, the assay was carried out in black flat bottom 96-well plates using a reaction volume of 100 µL. The phosphatases (PTP1B or TC-PTP) were preincubated with the tested compounds (0.1–80 µM) in 50 mM HEPES (pH 6.9), 100 mM NaCl, 1 mM EDTA, 2 mM DTT, 0.1 mg/mL BSA for 5 min at 37 °C. The following concentrations of the compounds were studied: 0.1, 0.2, 0.5, 1, 2, 5, 10, 20, 40, and 80 μM. The reactions were initiated by the addition of the fluorogenic substrate DiFMUP and diluted in the assay buffer. Progress curves for hydrolysis reaction were obtained with 25 µM of DiFMUP for 80 ng/mL PTP1B, and 35 µM of DiFMUP for 80 ng/mL TC-PTP. Fluorescence excitation of hydrolyzed DiFMUP and the fluorescent standard DiFMU was monitored at 355 nm and emission was detected at 460 nm for 6–10 min at 30 s intervals in a Fluoroskan Ascent FL microplate reader (Thermo Electron Corporation, Vantaa, Finland). Initial velocities of the reactions were used to calculate the IC_50_ values using GraphPad Prism.

#### 4.2.2. Animals and the Induction of Obesity

The male Wistar rats (at the start of the experiment the age of the animals was 2-months, and at the end of the experiment their age was 6-months) were obtained from the Rappolovo animal facility (Russia). The animals were housed in plastic cages, five animals in each, with a normal light–dark cycle (12 h/12 h), a temperature of 22 ± 3 °C, and free access to food and water. All experiments were approved by the Institutional Animal Care and Use Committee at the Sechenov Institute of Evolutionary Physiology and Biochemistry (St. Petersburg, Russia) (protocol No 02/02-2020, 19 February 2020) and according to The Guide for the Care and Use of Laboratory Animals and the European Communities Council Directive of 1986 (86/609/EEC). All efforts were made to minimize animal suffering and reduce the number of experimental animals.

The obesity model was induced at 16-weeks (starting at two months of age) using a high-fat diet. Control animals received standard laboratory chow pellets. The high-fat diet included supplements of 5–7 g of a fat mixture containing 52.4% pork lard, 41.7% curd, 5% liver, 0.5% *L*-methionine, 0.2% baker’s yeast, and 0.2% sodium chloride [34]. After 16-weeks on a high-fat diet, the animals with increased body weight, glucose intolerance, and hyperinsulinemia, and hyperleptinemia were selected for further experiments. The glucose intolerance was estimated according to the results of the glucose tolerance test (GTT). In the GTT, 120 min after glucose loading, the blood glucose levels in obese rats were above 7 mM, and the AUC_0-120_ values for the curve “glucose concentration (mM)–time (minutes)” were above 30%, as compared to the average AUC_0-120_ values in the control group.

Further, six groups were formed: control (Con, n = 10), obesity (Ob, n = 10), obese rats with five-day treatment with compounds **1** (Ob + **1**, n = 5), **2** (Ob + **2**, n = 5), **3** (Ob + **3**, n = 10), and **4** (Ob + **4**, n = 10). All compounds were administered in DMSO (300 µL) at a daily dose of 7 mg/kg (**1** and **2**), 8 mg/kg (**3**), and 10 mg/kg (**4**) (i.p.), based on the results of the preliminary experiments. The used doses of compounds **1**–**4**, in terms of the number of moles of each compound per kg of animal body weight, were equivalent. Control and obese rats received DMSO in the same volumes (300 µL), instead of the tested compounds. During the five days of the experiment, all animals were transferred to standard food and had free access to food and drinking water. Five animals each from the control group and the groups with obesity and treated with the most active compounds **3** and **4** were selected for the study of glucose tolerance using the GTT. The test was carried out on the morning of the next day after the last (fifth) injection of the compounds or DMSO, after a 10 h fast. The rest of the animals (n = 5 in each group) were anesthetized and decapitated on the last day of the experiment. The blood and liver samples were taken to measure blood glucose and hormone levels and gene expression in the liver.

#### 4.2.3. The Determination of Blood Glucose and Hormones Levels and GTT

The glucose levels in the blood obtained from the tail vein were measured using a glucometer (Life Scan Johnson & Johnson, Denmark) and the test strips “One Touch Ultra” (USA). The levels of insulin and leptin in rat serum were estimated with the “Rat Insulin ELISA” (Mercodia AB, Uppsala, Sweden) and “ELISA for Leptin, Rat” (Cloud-Clone Corporation, Houston, TX, USA) kits. The GTT was carried out using a single injection of glucose (2 g/kg, i.p.) after 10 h of fasting, as described earlier [34]. The blood glucose levels were measured before (0 min) and 15, 30, 60, and 120 min after the glucose load.

#### 4.2.4. The Determination of Gene Expression in the Liver of Rats

The total RNA was isolated from the liver samples of rats using the “ExtractRNA Reagent” (Evrogen, Moscow, Russia), and the samples containing 1 μg of RNA were transcribed to cDNA using the random oligodeoxynucleotide primers and the “MMLV RT kit” (Evrogen, Russia). The amplification procedure was carried out in the mixture containing 10 ng of reverse transcribed product, 0.4 μM of the forward and reverse primers, and the “qPCRmix-HS SYBR + LowROX kit” (Evrogen, Russia). The amplified signals were detected using the “Applied Biosystems^®^ 7500 Real-Time PCR System” (Life Technologies, Carlsbad, CA, USA, Thermo Fisher Scientific Inc., USA). The primers that were used to assess the expression of genes encoding the phosphatase PTP1B, TC-PTP, and SHP1 and the insulin and leptin receptors are presented in Table 4. The obtained data were calculated with the delta–delta C_t_ method and expressed as a fold expression, relative to the corresponding control [35]. The expression of the gene encoding 18S RNA was used as an endogenous control.

#### 4.2.5. Statistical Analysis of Biological Experiments

The data on food intake, body weight, and biochemical parameters in rats, as well as the PCR data, were analyzed using the IBM SPSS Statistics 22 software (“IBM”, Armonk, NY, USA), and the results are presented as mean ± standard error of the mean (*M ± SEM*). All differences are considered significant at *p* < 0.05. The calculation of the IC_50_ values of the studied inhibitors for the initial velocities of enzymatic reactions (PTP1B, TC-PTP) was carried out using the nonlinear regression analysis with GraphPad Prism 8 (“GraphPad Software, Inc.”, Boston, MA, USA). The statistical analysis was carried out using the Wilcoxon test for pairwise comparison and Dunn’s test for multiple comparisons.

### 4.3. Computational Details

#### 4.3.1. Used Enzyme Models

Observed protein structures were taken from the RCSB Protein Data Bank database [36]. PDB IDs: 1Q1M (PTP1b), 1L8K (TC-PTP), 4GRZ (SHP1).

#### 4.3.2. Protein and Ligand Structure Preparation

All proteins (enzymes) were preprocessed before the calculations using the protein prep wizard tool from Schrodinger suite 2021-4 [37]. During preprocessing, the following errors were fixed: missing amino acid sidechains, incorrect protonation states, missing hydrogens, incorrect bond orders, bond angles, bond length, and torsion angles. Solvent molecules were removed from all protein structure models.

The three-dimensional structure of the compounds was generated using the LigPrep module with the computation of possible ionization states, tautomers, and stereoisomers at the physiological pH (7.4)

All manipulations with the protein structures and small molecules were performed in the OPLS4 forcefield.

#### 4.3.3. Molecular Docking and MM-GBSA

All protein models (PTP1B, TC-PTP, and SHP1) were superimposed with the use of the protein to PTP1B structure, using the protein alignment tool (PTP1B was used as the reference). The active site was defined by positioning the reference ligand, which was present in the PTP1B model (PDB id: 1Q1M). The grid box was placed in accordance with the centroid of the workspace ligand structure. The grid box side size was 12 Å (in accordance with ligand size and the addition of the non-bonded interactions distance). The scaling factor was 1.0; the partial charge cutoff was 0.25, without the excluded volumes.

The reference structure and the other studied compounds were docked in the PTP1B, TC-PTP, and SHP1 active sites. The Glide program [38] included in Schrodinger Suite was used for the docking. For each ligand, 15 docking solutions in standard precision (SP) mode were generated, without the use of constraints. Best-fitting binding pose was selected by comparing ligand–protein interactions with those for the reference ligand (see Figure 9). For each docked structure a ligand interaction diagram was generated, describing the protein–ligand contacts and types.

Gibbs free energy was calculated with the use of the MM–GBSA method [39], including the implicit solvent model. This method considers the influence of the solvent and analyzes the free energy components, such as the energy increments of the strained contacts, solvation energy, and the ligand-induced conformational changes in protein amino acids surrounding the active site that interact with the ligand.

For calculations, the best protein–ligand complexes obtained by docking were used. The VSGB solvation model was used along with the OPLS4 forcefield. Protein flexibility was allowed at a distance of 6 Å from the ligand. The calculations were performed with the use of the Prime module [40] from Schrodinger Suite 2021-4.

## 5. Conclusions

We have studied the inhibitory effects of a small series of 4-oxo-1,4-dihydrocinnolines **1**–**4** in vitro, which exhibited dual inhibitory profiles towards two phosphatases, PTP1B and TC-PTP, which are both involved in insulin and leptin signaling. While two compounds (**1** and **2**) demonstrated different selectivity for the studied tyrosine phosphatases, PTP1B and TC-PTP, the selectivity of compounds **3** and **4** for these phosphatases was comparable. The affinity of the compounds to both phosphatases was corroborated by in silico modeling experiments. In obese rats, compounds **3** and **4** restored metabolic and hormonal parameters to a greater extent than compounds **1** and **2**, which were more selective for PTP1B than TC-PTP. Thus, the development of phosphatase inhibitors with similar selectivity for PTP1B and TC-PTP may become a promising direction for the development of drugs for the correction of hyperphagia and obesity and the treatment of metabolic disorders, such as type 2 diabetes mellitus and metabolic syndromes.

## Figures and Tables

**Table 1:** The IC_50_ values (μM) for the inhibitory effects of compounds **1–4** on PTP1B and TC-PTP activities.

Compound	PTP1B	TC-PTP
**1**	3.78 ± 0.34	18.87 ± 2.06 ^b^
**2**	6.01 ± 0.59	42.79 ± 9.93 ^a,b^
**3**	5.11 ± 0.57	2.98 ± 0.76
**4**	11.04 ± 1.34	26.54 ± 4.66

^a^: the difference between PTP1B and TC-PTP is significant at *p* < 0.05; ^b^: the difference between compound **3** and other compounds (IC_50_ for TC-PTP) is significant at *p* < 0.05. *M ± SEM* (n = 24).

**Table 2:** Effect of the five-day administration of compounds **1–4** on the blood glucose, insulin and leptin levels and the insulin resistance index in obese rats before and after drug treatments, as compared with control animals.

Group	Glucose, mM		Insulin, ng/mL		IR, arb.Units		Leptin, ng/mL	
	Before	After	Before	After	Before	After	Before	After
Control	4.40 ± 0.15	4.24 ± 0.13	0.66 ± 0.07	0.64 ± 0.12	2.93 ± 0.38	2.73 ± 0.55	1.09 ± 0.12	1.06 ± 0.22
Obese	5.86 ± 0.13 ^a^	5.72 ± 0.19 ^a^	1.62 ± 0.11 ^a^	1.49 ± 0.18 ^a^	9.52 ± 0.75 ^a^	8.49 ± 1.07 ^a^	3.04 ± 0.29 ^a^	2.68 ± 0.26 ^a^
Ob + **1**	5.94 ± 0.22 ^a^	5.74 ± 0.07 ^a^	1.57 ± 0.16 ^a^	1.11 ± 0.14 ^a^	9.46 ± 1.31 ^a^	6.32 ± 0.77 ^a^	3.06 ± 0.40 ^a^	1.72 ± 0.24 ^b,c^
Ob + **2**	5.82 ± 0.22 ^a^	5.68 ± 0.09 ^a^	1.65 ± 0.17 ^a^	1.19 ± 0.13 ^a,c^	9.68 ± 1.27 ^a^	6.79 ± 0.76 ^a^	3.27 ± 0.38 ^a^	2.18 ± 0.22 ^a,c^
Ob + **3**	5.80 ± 0.31 ^a^	5.36 ± 0.31 ^a^	1.51 ± 0.18 ^a^	0.93 ± 0.13 ^b,c^	8.96 ± 1.46 ^a^	5.12 ± 0.97 ^b^	2.93 ± 0.42 ^a^	1.52 ± 0.21 ^b,c^
Ob + **4**	5.72 ± 0.30 ^a^	5.24 ± 0.25 ^a^	1.65 ± 0.18 ^a^	1.06 ± 0.13 ^a,c^	9.63 ± 1.57 ^a^	5.61 ± 0.89 ^a^	3.17 ± 0.25 ^a^	1.90 ± 0.16 ^a,b,c^

*Note*. All compounds were administered in DMSO (300 µL) at a daily dose of 7 mg/kg (**1** and **2**), 8 mg/kg (**3**), and 10 mg/kg (**4**) (i.p.). ^a^: the difference to the control is significant at *p* < 0.05; ^b^: the difference to the untreated obese rats (Ob) is significant at *p* < 0.05; ^c^: the difference between the values, before and after treatment, with compounds **1**–**4** or DMSO. The data are presented as *M ± SEM*, n = 5.

**Table 3:** Docking and MM-GBSA ΔG free energy results; GScore: the combined value of GlideScore with penalties; LE: ligand efficiency (GlideScore divided by the number of non-hydrogen atoms).

Structure	Scores (kcal/mol)								
	PTP1B			TC-PTP			SHP1		
	GScore	MMGBSA ΔG	LE	GScore	MMGBSA ΔG	LE	GScore	MMGBSA ΔG	LE
ref.	−8.36	−83.78	0.28	-	-	-	-	-	-
**1**	−6.02	−41.48	0.46	−4.93	−31.28	0.38	−4.36	−23.80	0.33
**2**	−6.40	−41.43	0.46	−4.78	−33.08	0.34	−4.19	−21.61	0.30
**3**	−5.94	−43.74	0.42	−4.60	−40.06	0.33	−3.76	−18.06	0.26
**4**	−5.59	−44.17	0.40	−3.98	−38.42	0.33	−3.77	−18.29	0.27

**Table 4:** The structure of primers that were used for gene expression study.

Genes	Forward/Reverse Sequence	Product Size (bp)	Annealing Temperature (°C)	Genbank
*PTP1B*	(For) CAACCGAGGAGGAACAAAAGG	129	57	NM_012637
	(Rev) CAGTCTGTCAGTGAAAACATACCCG			
*TCPTP*	(For) GTGGAAGAGAGCAGTGAGAGT	216	56	NM_053990
	(Rev) GGCTTCAGGTCTTGCTTTGC			
*InsR*	(For) CTGGAGAACTGCTCGGTCATT	139	56	NM_017071
	(Rev) GGCCATAGACACGGAAAAGAAG			
*LepR*	(For)GCATGCAGAATCAGTGATATTTGG	81	57	NM_012596
	(Rev) CAAGCTGTATCGACACTGATTTCTTC			
*Shp1*	(For) CAGACTGTTCCCTCCACGTTT	193	56	NM_053908
	(Rev) TGGTCATCCACCCTGACTGA			
*18S*	(For) GGACACGGACAGGATTGACA	50	56	NR_046237
	(Rev) ACCCACGGAATCGAGAAAGA			

## Data Availability

Not applicable.
